# Associations between body composition and hospitalization in patients undergoing maintenance hemodialysis

**DOI:** 10.1111/ggi.70167

**Published:** 2025-09-06

**Authors:** Hae Eun Jeon, Hongtae Kim, Soie Kwon, Jungho Shin

**Affiliations:** ^1^ Department of Internal Medicine Chung‐Ang University Hospital Seoul South Korea

**Keywords:** body composition, hospitalization, maintenance hemodialysis, nutrition, volume status

## Abstract

**Aim:**

Patients undergoing maintenance hemodialysis (MHD) often have multiple comorbidities and are vulnerable to minor stressors that frequently result in hospitalization. Recent advances have enabled the easy estimation of body composition in clinical settings. This study retrospectively investigated changes in body composition associated with hospitalization in patients receiving MHD.

**Methods:**

Body composition was measured every 6 months using a multifrequency bioelectrical impedance analysis (BIA) device in outpatients with MHD. Hospitalization events lasting >24 h were reviewed throughout the follow‐up period.

**Results:**

Overall, 166 patients (94 men [56.6%]) underwent 904 BIA measurements. A higher baseline extracellular water to total body water ratio (ECW/TBW) and lower phase angle (PhA) were associated with increased hospitalization risk. During the study period, 272 hospitalizations were recorded. Compared with those without hospitalization, hospitalized patients showed progressive declines in fat‐free mass index, skeletal muscle mass index, and PhA, along with an increase in ECW/TBW. These trends were consistent across subgroups based on the number and duration of hospitalizations; however, an increase in visceral fat area was observed in patients with four or more hospitalizations. Among the 89 patients who were hospitalized between measurements, body composition was compared pre‐ and post‐hospitalization, with the results revealing significant decreases in body mass index, percent body fat, and PhA, and an increase in ECW/TBW.

**Conclusions:**

Body composition is closely associated with hospitalization in patients undergoing MHD. Efforts to maintain euvolemic status and active interventions such as nutritional support and rehabilitation therapy may be essential to preserve a favorable body composition in this vulnerable population. **Geriatr Gerontol Int 2025; 25: 1389–1396**.

## Graphical abstract

Hospitalization was associated with decreased muscle mass and phase angle and with volume overload. Strategies such as volume control, rehabilitation, and nutritional support may help preserve body composition in patients undergoing maintenance hemodialysis.

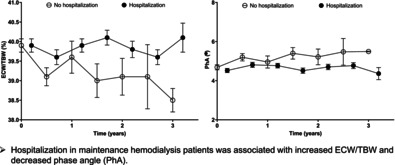

## Introduction

Patients with chronic kidney disease are at high risk of hospitalization and death, with odds increasing exponentially as kidney function declines.^1^, ^2^ Individuals with end‐stage kidney disease undergoing maintenance hemodialysis (MHD) often have multiple comorbidities and are highly susceptible to clinical deterioration, even from minor stressors. In this population, the risk of hospitalization is elevated, with hospital stays being prolonged and high readmission rates.^3^ Hospitalization itself is a known precursor to disability,^4^ and early hospital readmission is a predictor of subsequent mortality in patients receiving MHD.^5^ Moreover, frequent and prolonged hospitalizations place a substantial burden on healthcare systems, accelerating expenditures for managing this disease population.^6^ Considering the growing burden, research is essential for identifying risk factors for hospitalization and developing strategies for prevention and recovery promotion.

Recent advances in body composition assessment technologies have enhanced the ability to detect and diagnose individuals at risk of sarcopenia and malnutrition. However, evidence regarding the relationship between body composition parameters and hospitalization remains limited. Previous studies assessing body composition have reported that low muscle and fat masses are associated with high mortality in patients undergoing MHD.^7^, ^8^ Hypervolemia is prevalent in this population and increases cardiovascular morbidity and mortality.^9^ Nonetheless, the role of body composition parameters in predicting hospitalization remains unclear. Conversely, hospitalization has adverse effects on muscle mass and function, largely due to bed rest and physical inactivity.^10^, ^11^ Another study reported that acute inflammation is associated with low muscle mass and function in hospitalized male patients.^12^ These changes manifest as acute sarcopenia and contribute to poor recovery and adverse outcomes.^13^ Understanding how body composition changes in response to hospitalization may offer insights into early risk identification and guide interventions to preserve nutritional and functional status.

Therefore, this retrospective study investigated the longitudinal relationship between body composition and hospitalization in patients undergoing MHD. We examined body composition parameters associated with hospitalization and assessed both short‐ and long‐term changes in body composition in response to hospitalization using serial bioelectrical impedance analysis (BIA) measurements. Our findings support the need for clinical strategies to mitigate the adverse effects of hospitalization and promote recovery in this high‐risk population.

## Material and methods

### Patients

This study recruited 180 adults (≥18 years) with end‐stage kidney disease undergoing outpatient MHD who had at least one body composition measurement between October 2016 and October 2023. Among eligible individuals, 14 were excluded for the following reasons: measurements obtained during hospitalization (*n* = 7), absence of baseline laboratory data (*n* = 5), and presence of major limb amputation (*n* = 2). Overall, 166 patients were included and followed up until loss to follow‐up, kidney transplantation, conversion to peritoneal dialysis, death, or study endpoint (April 2024). This study was conducted in accordance with the Declaration of Helsinki and approved by the Institutional Review Board (IRB) of Chung‐Ang University Hospital (IRB number: 2310‐022‐19 496). The requirement for obtaining written consent was waived because the patients were anonymized owing to the retrospective nature of this study.

### Data collection

Demographic and clinical data, including age, sex, MHD duration, cause of end‐stage kidney disease, and comorbidities, was obtained from electronic medical records. The comorbidity burden was assessed using the modified Charlson Comorbidity Index.^14^ Additionally, drugs used during the study periods and those affecting body weight, fat distribution, and metabolic function, such as glucocorticoids, insulin, thiazolidinedione, antipsychotics, antiseizures, antidepressants, lithium, and β‐blockers, were reviewed.^15^ Fasting blood samples were collected before midweek dialysis sessions and included hemoglobin, albumin, urea nitrogen, creatinine, calcium, phosphorus, uric acid, sodium, potassium, total carbon dioxide, total cholesterol, high‐sensitivity C‐reactive protein, and intact parathyroid hormone levels. Post‐dialysis urea nitrogen levels were also measured. Dialysis adequacy (Kt/Vurea) and normalized protein catabolic rates were calculated using a single‐pool urea kinetic model.^16^

### Body composition analysis

Body composition was assessed every 6 months using a segmental multifrequency BIA device (InBody S10; BioSpace, Seoul, South Korea) after midweek dialysis sessions. Eight touch‐type electrodes were placed on the tips of the thumbs and middle fingers of both hands and between the malleoli and calcanei of both feet in the supine position. The BIA device was regularly calibrated and operated by trained nursing staff according to the manufacturer's instructions (http://www.inbody.com).

The obtained parameters included body weight, fat‐free mass, skeletal muscle mass, percent body fat (PBF), visceral fat area (VFA), ratio of extracellular water to total body water (ECW/TBW), and phase angle (PhA). Body mass index (BMI), fat‐free mass index (FFMI), and skeletal muscle mass index (SMI) were calculated by dividing body weight, fat‐free mass, and skeletal muscle mass by the square of height (kg/m^2^), respectively.

### Hospitalization

All hospitalizations during the study period were reviewed using electronic medical records. The following events were excluded: hospital stays of <24 h, admissions for diagnostic examinations, and hospitalizations for kidney transplantation. Each event was classified based on its etiology: cardiovascular disease, infection, other medical causes, dialysis access‐related problems, other surgical causes, malignancy, trauma, or degenerative diseases. The number of hospitalizations was categorized into four groups: none, one, two–three, and four or more, and the duration of hospitalization was categorized as 0, 1–14, 15–30, and >30 days. Additionally, hospitalizations requiring intensive care unit (ICU) admission were identified.

### Outcomes

We investigated the effect of body composition parameters on the risk of hospitalization. Then, we evaluated the impact of hospitalization on body composition using longitudinally measured BIA data. The trajectories of body compositions were compared according to the event and number of hospitalizations. We further assessed changes in body composition parameters before and after the first hospitalization event that occurred between BIA measurements. For comparison, we also considered whether admission to the general ward or ICU was required.

### Statistical analysis

Continuous variables were expressed as means ± standard deviation, and categorical variables were expressed as counts and percentages. Cox proportional hazards regression was conducted to estimate the impact of body composition on future hospitalization. Hazard ratios (HRs) and 95% confidence intervals (CIs) for predicting hospitalization were estimated. Linear mixed‐effects models were used to evaluate longitudinal changes in body composition according to hospitalization status. Additionally, paired *t*‐tests were used to compare parameters pre‐ and post‐hospitalization. Multivariate analyses were performed after adjusting for age, sex, Charlson Comorbidity Index, high‐sensitivity C‐reactive protein level, and glucocorticoid use in both the Cox regression and linear mixed‐effect models. These variables were selected based on univariate associations (Fig. S1). All statistical analyses were performed using R software (version 4.4.2; The R Foundation for Statistical Computing, Vienna, Austria). Statistical significance was defined as a two‐sided *P*‐value of <0.05.

## Results

### Baseline characteristics

This study included a total of 166 patients (94 [56.6%] men and 72 [43.4%] women) with a mean age of 68 ± 13 years. Diabetes was present in 95 (57.2%) patients, and their modified Charlson Comorbidity Index was 2.9 ± 2.2. Baseline characteristics, including laboratory results and body composition parameters, are summarized in Table 1. Correlations between body composition parameters and clinical variables, including age, sex, Charlson Comorbidity Index, high‐sensitivity C‐reactive protein level, and medication use, were assessed, and several significant associations were identified (Fig. S1).

**Table 1:** Baseline characteristics of patients undergoing MHD

Variable	Total (*n* = 166)
Age, years	68 ± 13
Males, n (%)	94 (56.6)
MHD duration, months	41 ± 57
Diabetes, n (%)	95 (57.2)
Charlson Comorbidity Index	2.9 ± 2.2
Drugs, n (%)	
Glucocorticoids	19 (11.4)
Insulin	32 (19.3)
Thiazolidinedione	1 (0.6)
Antipsychotics	11 (6.6)
Antiseizures	22 (13.3)
Antidepressants	20 (12.0)
Lithium	0 (0)
β‐blockers	90 (54.2)
Ultrafiltration rate, mL/kg/h	7.0 ± 3.5
Laboratory	
Hemoglobin, g/dL	10.2 ± 1.5
Albumin, g/dL	3.6 ± 0.4
Urea nitrogen, mg/dL	51.3 ± 14.3
Creatinine, mg/dL	7.4 ± 2.3
Calcium, mg/dL	8.8 ± 0.9
Phosphorus, mg/dL	4.6 ± 1.4
Uric acid, mg/dL	6.0 ± 1.4
Sodium, mmol/L	135.9 ± 3.4
Potassium, mmol/L	4.5 ± 0.7
Total carbon dioxide, mmol/L	25.1 ± 2.7
Total cholesterol, mg/dL	142.9 ± 39.1
High‐sensitivity C‐reactive protein, mg/L	8.9 ± 19.2
Intact parathyroid hormone, pg/mL	270.8 ± 220.7
Kt/V_urea_	1.7 ± 0.3
Normalized protein catabolic rate, g/kg/day	1.0 ± 0.2
Body composition	
BMI, kg/m^2^	22.4 ± 3.5
FFMI, kg/m^2^	16.5 ± 2.3
SMI, kg/m^2^	9.5 ± 1.3 (men); 7.9 ± 1.0 (women)
PBF, %	25.4 ± 10.6
VFA, cm^2^	75.5 ± 46.3
ECW/TBW, %	39.9 ± 1.5
PhA, °	4.6 ± 1.1
Hospitalization, *n* (%)	115 (69.3)
Death, *n* (%)	35 (21.1)

*Note*: Data are expressed as mean ± standard deviation or numbers (percentage).

Abbreviations: BMI, body mass index; ECW/TBW, ratio of extracellular water to total body water; FFMI, fat‐free mass index; PBF, percent body fat; PhA, phase angle; SMI, skeletal muscle mass index; VFA, visceral fat area.

### Impact of body composition on hospitalization

Hospitalization occurred in 115 (69.3%) patients, and the time to first hospitalization was 15 ± 21 months. Associations between baseline body composition and hospitalization were identified (Table 2). High ECW/TBW and low PhA predicted the incidence of hospitalization, independent of age, sex, and Charlson Comorbidity Index (HR: 1.2 [95% CI: 1.1–1.4] and 0.6 [95% CI: 0.5–0.8]; *P* = 0.001 and *P* < 0.001). Additionally, the time to admission for the two most common reasons, infection and cardiovascular disease, was evaluated, and similar results were observed, despite some trends of SMI and VFA with infection requiring hospitalization.

**Table 2:** Impact of body composition on hospitalization in patients undergoing MHD

Variable	Any hospitalization		Infection		Cardiovascular disease	
	HR (95% CI)	*P*	HR (95% CI)	*P*	HR (95% CI)	*P*
BMI, kg/m^2^	1.0 (0.9–1.0)	0.514	1.0 (1.0–1.1)	0.405	1.0 (0.9–1.1)	0.661
FFMI, kg/m^2^	1.0 (0.9–1.1)	0.701	0.9 (0.8–1.1)	0.343	1.1 (0.9–1.4)	0.387
SMI, kg/m^2^	0.9 (0.8–1.1)	0.484	0.9 (0.7–1.1)	0.239	1.1 (0.8–1.6)	0.633
PBF, %	1.0 (1.0–1.0)	0.505	1.0 (1.0–1.1)	0.115	1.0 (0.9–1.0)	0.214
VFA, cm^2^	1.0 (1.0–1.0)	0.638	1.0 (1.0–1.0)	0.072	1.0 (1.0–1.0)	0.736
ECW/TBW, %	1.2 (1.1–1.4)	0.001	1.3 (1.0–1.6)	0.019	1.5 (1.2–1.8)	0.001
PhA, °	0.6 (0.5–0.8)	<0.001	0.4 (0.3–0.7)	<0.001	0.5 (0.3–0.9)	0.013

*Note*: Multivariate analysis was adjusted for age, sex, the modified Charlson Comorbidity Index, high‐sensitivity C‐reactive protein level, and glucocorticoid use.

Abbreviations: BMI, body mass index; CI, confidence interval; ECW/TBW, ratio of extracellular water to total body water; FFMI, fat‐free mass index; HR, hazard ratio; PBF, percent body fat; PhA, phase angle; SMI, skeletal muscle mass index; VFA, visceral fat area.

### Body composition changes by hospitalization

All included patients underwent 904 BIA measurements throughout the study period. Between measurement intervals, 272 hospitalization events were recorded. The most common cause was infection (37.1%), followed by cardiovascular disease (22.1%), other medical causes (17.3%), dialysis access‐related problems (5.9%), other surgical causes (5.9%), malignancies (5.5%), trauma (4.0%), and degenerative conditions (4.0%) (Fig. S2).

**Figure 1:**
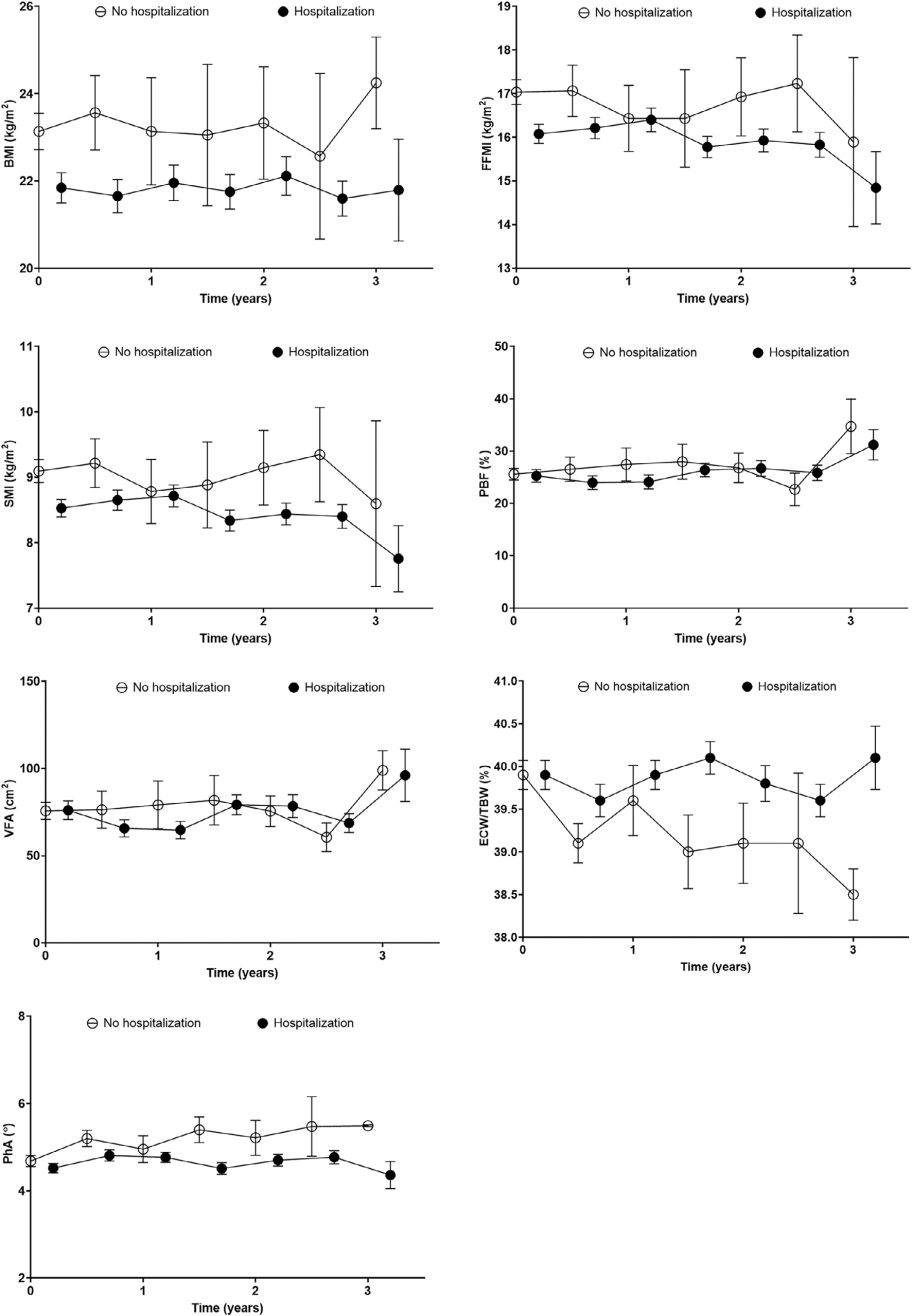
Changes in body composition parameters according to hospitalization. (a, d, e) Slopes of BMI, PBF, and VFA did not differ between patients with and without hospitalization events (*P* = 0.929). (b, c, g) Hospitalized patients exhibited declining FFMI, SMI, and PhA values (−0.2 [−0.3 to −0.0] kg/m^2^ per year, −0.1 [−0.2, −0.0] kg/m^2^ per year, and −0.1° [−0.2° to −0.0°] per year; *P* = 0.040, 0.018, and 0.028). (f) Patients with hospitalization events presented with higher ECW/TBW over time compared with those without events (0.2 [0.0 to 0.3]% per year; *P* = 0.005). Multivariate analysis was adjusted for age, sex, the modified Charlson Comorbidity Index, high‐sensitivity C‐reactive protein level, and glucocorticoid use. BMI, body mass index; ECW/TBW, ratio of extracellular water to total body water; FFMI, fat‐free mass index; PBF, percent body fat; PhA, phase angle; SMI, skeletal muscle mass index; VFA, visceral fat area.

The trajectories of body compositions were compared based on hospitalization history (Fig. 1). Annual declines in FFMI, SMI, and PhA were greater in patients who experienced hospitalization than in those who did not (−0.2 [95% CI: −0.3 to −0.0] kg/m^2^ per year, −0.1 [95% CI: −0.2 to −0.0] kg/m^2^ per year, and − 0.1° [95% CI: −0.2° to −0.0°]; *P* = 0.040, 0.018, and 0.028, respectively). Conversely, ECW/TBW accumulated following hospitalization (0.2% [95% CI: 0.0–0.3%] per year; *P* = 0.005). No significant associations were found between BMI, PBF, and VFA (*P* = 0.939, 0.585, and 0.228, respectively).

We further evaluated the effect of the number and duration of hospitalizations on changes in body composition (Table 3). FFMI, SMI, and PhA decreased, and ECW/TBW increased over time in hospitalized patients; however, these changes were not proportional to event frequency and duration. However, an increase in VFA was observed in patients who were admitted four or more times.

**Table 3:** Body composition changes according to the hospitalization number and duration

Variable	Hospitalization frequency		Hospitalization duration	
	Number (*n*)	Estimate (95% CI)	Duration (days)	Estimate (95% CI)
BMI (kg/m^2^ per year)	1 2–3 ≥4	−0.1 (−0.4 to 0.2) −0.0 (−0.4 to 0.3) 0.1 (−0.2 to 0.4)	1–14 15–30 >30	−0.1 (−0.3 to 0.2) −0.0 (−0.4 to 0.3) 0.1 (−0.2 to 0.3)
FFMI (kg/m^2^ per year)	1 2–3 ≥4	−0.1 (−0.2 to 0.1) −0.2 (−0.4 to −0.0)* −0.2 (−0.4 to −0.0)*	1–14 15–30 >30	−0.1 (−0.3 to 0.1) −0.3 (−0.4 to −0.1)* −0.2 (−0.3 to 0.0)
SMI (kg/m^2^ per year)	1 2–3 ≥4	−0.1 (−0.2 to 0.0) −0.1 (−0.2 to −0.0)* −0.1 (−0.2 to −0.0)*	1–14 15–30 >30	−0.1 (−0.2 to 0.0) −0.2 (−0.3 to −0.1)* −0.1 (−0.2 to −0.0)*
PBF (% per year)	1 2–3 ≥4	−0.4 (−1.3 to 0.5) 0.2 (−0.7 to 1.2) 0.7 (−0.2 to 1.6)	1–14 15–30 >30	−0.1 (−1.1 to 0.9) 0.6 (−0.4 to 1.6) 0.4 (−0.6 to 1.3)
VFA (cm^2^ per year)	1 2–3 ≥4	−0.4 (−3.7 to 3.0) 1.8 (−1.7 to 5.3) 3.7 (0.4 to 7.0)*	1–14 15–30 >30	0.9 (−2.6 to 4.3) 3.3 (−0.5 to 7.1) 2.2 (−1.4 to 5.7)
ECW/TBW (% per year)	1 2–3 ≥4	0.1 (0.0 to 0.3)* 0.2 (0.1 to 0.3)* 0.1 (0.0 to 0.3)*	1–14 15–30 >30	0.1 (0.0 to 0.3)* 0.2 (0.1 to 0.3)* 0.2 (0.0 to 0.3)*
PhA (° per year)	1 2–3 ≥4	−0.1 (−0.2 to 0.0)* −0.1 (−0.2 to −0.0)* −0.1 (−0.2 to 0.0)*	1–14 15–30 >30	−0.1 (−0.2 to 0.0) −0.1 (−0.2 to −0.0)* −0.1 (−0.2 to 0.0)

*Note*: Multivariate analysis was adjusted for age, sex, the modified Charlson Comorbidity Index, high‐sensitivity C‐reactive protein, and glucocorticoid use. Estimates (95% CI) were calculated and compared with those for patients without hospitalization.

* *P* < 0.05.

Abbreviations: BMI, body mass index; CI, confidence interval; ECW/TBW, ratio of extracellular water to total body water; FFMI, fat‐free mass index; PBF, percent body fat; PhA, phase angle; SMI, skeletal muscle mass index; VFA, visceral fat area.

### Body composition changes following hospitalization

**Figure 2:**
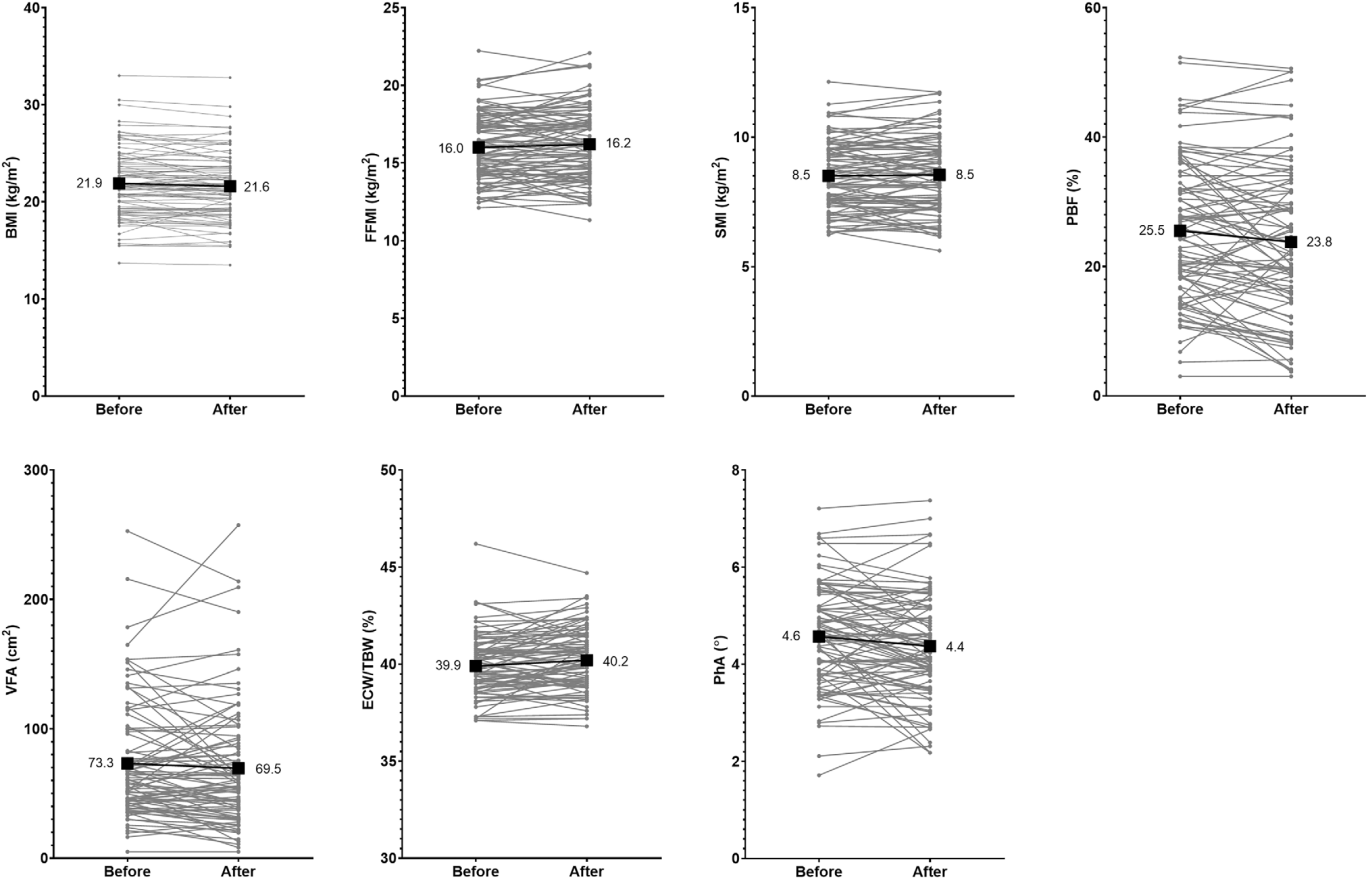
Body composition parameters before and after hospitalization. There were 89 patients who were admitted between BIA measurements. (a, d) BMI and PBF significantly decreased by 0.3 ± 1.1 kg/m^2^ and 1.8 ± 6.2% (*P* = 0.007 and 0.009). (b, c, e) FFMI, SMI, and VFA did not change according to hospitalization. (f, g) ECW/TBW increased by 0.3 ± 1.1%, while PhA decreased by 0.2° ± 0.7° (*P* = 0.009 and 0.013) following hospitalization. BIA, bioelectrical impedance analysis; BMI, body mass index; ECW/TBW, ratio of extracellular water to total body water; FFMI, fat‐free mass index; PBF, percent body fat; PhA, phase angle; SMI, skeletal muscle mass index; VFA, visceral fat area.

Body composition parameters were compared pre‐ and post‐hospitalization in the 89 patients admitted between regular BIA measurements (Fig. 2). Both BMI and PBF decreased following hospitalization (−0.3 ± 1.1 kg/m^2^ and − 1.8% ± 6.2%; *P* = 0.007 and 0.009, respectively). In contrast, no changes were observed in FFMI, SMI, or VFA (*P* = 0.268, 0.590, and 0.177, respectively). Hospitalization was associated with an increase in ECW/TBW by 0.3 ± 1.1% and with a decrease in PhA by 0.2° ± 0.7° (*P* = 0.009 and 0.013, respectively).

Changes in body composition were further evaluated according to the type of admission, either to the general ward or to the ICU (Table S1). Significant reductions in BMI and PBF were noted among patients admitted to the general ward (*P* = 0.015 and 0.038, respectively); however, these changes were not observed among those admitted to the ICU (*P* = 0.252 and 0.115, respectively). Conversely, the impact of hospitalization on ECW/TBW and PhA was greater after ICU admission (*P* = 0.033 and 0.029, respectively).

## Discussion

This study retrospectively investigated the relationship between body composition and hospitalization in patients undergoing MHD. We evaluated the effect of body composition on the risk of hospitalization and found that high ECW/TBW and low PhA were associated with an increased risk. Conversely, compared with patients without hospitalization, those hospitalized during the study period had reduced FFMI, SMI, and PhA and increased ECW/TBW. Comparisons of body composition parameters pre‐ and post‐hospitalization revealed that BMI, PBF, and PhA decreased whereas ECW/TBW increased following the event. Therefore, a strong association exists between body composition and hospitalization.

Advances in techniques for assessing body composition have provided accessibility in clinical practice and gained attention for the clinical roles of body compositions in maintaining health status. Among various measures, BIA is a widely used and easily accessible device in clinical practice. Its accuracy, especially that of multifrequency devices, has been validated and recommended in clinical guidelines for malnutrition and sarcopenia.^17^, ^18^ Considering its relevance, we investigated the relationship between body compositions and hospitalization in patients undergoing MHD as well as aiming to identify strategies for maintaining healthy body content in these individuals. For each parameter reflecting muscle mass, fat mass, and volume status, the association with hospitalization was evaluated, and the findings are discussed. Additionally, we evaluated the role of PhA, which has been demonstrated as a useful indicator of nutritional status and inflammation and a predictor of prognosis.^19^, ^20^

Loss of muscle mass is prevalent in patients with chronic kidney disease, particularly in those with end‐stage kidney disease undergoing MHD, as a consequence of negative protein balance resulting from multiple etiologies, including aging, kidney disease, comorbidities, metabolic acidosis, chronic inflammation, and dialysis.^21^ Low muscle mass is a characteristic feature of sarcopenia and malnutrition^17^, ^22^ and it predicts adverse outcomes, including cardiovascular events and death.^23^, ^24^ In this study, we did not discover an impact of FFMI and SMI on the risk of hospitalization, despite the trend toward low SMI and an increased risk of hospitalization owing to infection. Conversely, a previous study showed that sarcopenia increased the incidence of pneumonia in older adults,^25^ and another study conducted at our center reported that low muscle mass (FFMI <17 kg/m^2^ for men and < 15 kg/m^2^ for women) increased the risk of infection requiring hospitalization in patients on MHD.^26^ Loss of muscle mass post‐hospitalization is problematic.^13^ Mechanisms underlying adverse effects on muscle mass and function appear to include the bed rest and physical inactivity associated with hospitalization.^10^, ^11^ Inflammation is another cause of sarcopenia following hospitalization.^12^ Consistent with these findings, this study demonstrated that the trajectories of FFMI and SMI significantly decreased in hospitalized patients compared with in those without the event. Therefore, efforts are required to prevent muscle wasting in hospitalized patients through nutritional support and exercise. Caution is required when interpreting these results because fluid retention can overestimate parameters, including BMI, FFMI, and SMI. Although BIA was performed after dialysis, the presence of edema could have influenced these parameters. ECW/TBW increased in hospitalized patients; thus, some reductions in BMI, FFMI, and SMI following hospitalization might have been overlooked.

Further, this study explored the association between fat content, as indicated by PBF and VFA, and hospitalization; however, we did not observe consistent connections in patients undergoing MHD. A reduction in PBF is a phenotype of protein‐energy wasting,^22^ and hospitalization may lead to a reduction in fat mass owing to undernutrition and inflammation. Few studies have investigated the association between fat content and hospitalization. Obesity is a risk factor for mortality in the general population; however, low fat and muscle masses are associated with higher mortality rates in patients undergoing MHD.^7^, ^8^ Conflicting results exist regarding the impact of sarcopenic obesity on outcomes in these patients.^27^, ^28^ Further research is necessary to understand accurately the role of fat and its interplay with muscle mass in adverse outcomes.

BIA is widely used for volume assessment, and its utility has been verified in individuals receiving MHD.^29^ An association between fluid overload assessed using BIA and adverse outcomes, including mortality and cardiovascular events, has been demonstrated.^30^, ^31^, ^32^ This study further revealed that fluid overload increased the risk of hospitalization, and notably, high ECW/TBW was associated with infections requiring hospitalization. However, this study found that patients who had any hospitalization event became fluid‐overloaded during the study period. Altogether, a vicious cycle may connect fluid overload and adverse outcomes. Accurate estimation of volume status is essential following hospitalization, and reactive adjustment of dry weight is required to prevent rehospitalization in vulnerable patients undergoing MHD, particularly those admitted to the ICU.

This study also observed a strong association between PhA and hospitalization in patients undergoing MHD. PhA is a derivative of BIA that indicates cell membrane integrity and cellular health.^33^ Various studies have verified its utility as an indicator of nutritional status, inflammation, and muscle quality and as a predictor of mortality in individuals receiving MHD.^20^, ^34^, ^35^, ^36^ Additionally, low PhA was found to associate with an increased risk of hospitalization, consistent with previous reports.^37^, ^38^ Moreover, hospitalization worsened PhA; thus, a decrease in PhA following hospitalization increased the risk of readmission. Therefore, PhA is an appropriate measure for monitoring patient vulnerability and a useful metric for assessing the effects of various interventions, such as nutritional support and exercise. Some studies have evaluated PhA changes in response to exercise, with positive results.^39^, ^40^, ^41^ More studies are needed to validate the utility of PhA in assessing the overall health of vulnerable individuals, such as those undergoing MHD.

This study had some limitations. First, this single‐center study with a small sample size may have limited power to detect significant differences. Indeed, changes in BIA parameters were disproportional to the number of hospitalizations, and some differences, such as PBF and VFA patterns according to hospitalization, were inconsistent. Second, the observational nature of this study may have introduced selection bias and overlooked some confounding variables. The limited number of covariates used for adjustment could also have contributed to bias. Third, the absence of subjective and objective measurements, such as muscle strength, physical function, frailty, or quality of life, could limit the significance of the study findings. These factors are strongly associated with both body composition and hospitalization. Fourth, this study could not confirm causality between body composition and hospitalization and did not consider the underlying mechanisms of the interplay between variables and outcomes. Additionally, this study could not determine the effects of interventions, such as rehabilitation and nutritional support, on body composition and hospitalization. We identified these values only as biomarkers. Therefore, more studies are required to understand the mechanisms underlying hospitalization risk based on body composition and pathological changes in response to hospitalization.

In conclusion, this study revealed the relationship between body composition and hospitalization in patients undergoing MHD. Volume overload and low PhA values increased the risk of hospitalization; conversely, hospitalization adversely affected body composition parameters, including muscle mass, fluid status, and PhA. Therefore, regular assessment of body composition can assist in estimating the risk of hospitalization, help assess the impact of hospitalization, and guide therapeutic interventions to preserve healthy body content.

## Disclosure statement

The authors declare no conflicts of interest.

## Author contributions

Hae Eun Jeon: conceptualization, data curation, formal analysis, and writing of the original draft. Hongtae Kim: data curation and investigation. Soie Kwon: data curation and supervision. Jungho Shin: conceptualization, data curation, formal analysis, investigation, supervision, and writing of the original draft.

## Ethics statement

This study was conducted in accordance with the Declaration of Helsinki and approved by the IRB of Chung‐Ang University Hospital (IRB number: 2310‐022‐19 496).

## Patient consent statement

The requirement for obtaining written consent was waived because the patients were anonymized owing to the retrospective nature of this study.

## Clinical trial registration

Not applicable.

## Supporting information


**Fig S1.** Correlation between clinical variables and body composition parameters. Color intensity indicates the strength of the correlation coefficient, with positive correlations shown in red and negative correlations in blue. *P < 0.05.
**Fig S2.** Distribution of hospitalization reasons in patients undergoing MHD. There were 272 hospitalization events between the BIA measurement intervals. The most common cause of admission was infection (*n* = 101 [37.1%]), followed by cardiovascular events (*n* = 60 [22.1%]), other medical causes (*n* = 47 [17.3%]), dialysis access‐related problems (*n* = 16 [5.9%]), other surgical causes (*n* = 16 [5.9%]), malignancy (*n* = 15 [5.5%]), trauma (*n* = 11 [4.0%]), and degenerative diseases (*n* = 6 [2.2%]). BIA, bioelectrical impedance analysis; MHD, maintenance hemodialysis.
**Table S1.** Body composition changes after hospitalization.

## Data Availability

Data supporting the findings of this study are available from the corresponding author upon request.
