# Supplementary material for: Associations between body composition and hospitalization in patients undergoing maintenance hemodialysis
Source: Geriatr Gerontol Int. 2025 Sep 6;25(10):1389–96. doi: 10.1111/ggi.70167 (PMC12501674; doi:10.1111/ggi.70167)
Supplement: Supplementary file 1 — Fig S1. Correlation between clinical variables and body composition parameters. Color intensity indicates the strength of the correlation coefficient, with positive correlations shown in red and negative correlations in blue. *P < 0.05. Fig S2. Distribution of hospitalization reasons in patients undergoing MHD. There were 272 hospitalization events between the BIA measurement intervals. The most common cause of admission was infection (n = 101 [37.1%]), followed by cardiovascular events (n = 60 [22.1%]), other medical causes (n = 47 [17.3%]), dialysis access‐related problems (n = 16 [5.9%]), other surgical causes (n = 16 [5.9%]), malignancy (n = 15 [5.5%]), trauma (n = 11 [4.0%]), and degenerative diseases (n = 6 [2.2%]). BIA, bioelectrical impedance analysis; MHD, maintenance hemodialysis. Table S1. Body composition changes after hospitalization. [file GGI-25-1389-s001.docx]

**Fig. S1.** Correlation between clinical variables and body composition parameters. Color intensity indicates the strength of the correlation coefficient, with positive correlations shown in red and negative correlation in blue. *P <0.05

BMI, body mass index; CCI, Charlson comorbidity index; CRP, C-reactive protein; ECW/TBW, extracellular water to total body water ratio; FFMI, fat-free mass index; GCS, glucocorticoid, PBF, percent body fat; PhA, phase angle; SMI, skeletal muscle index; TZD, thiazolidinedione; VFA, visceral fat area

**Fig. S2.** Distribution of hospitalization reasons in patients undergoing MHD. There were 272 hospitalization events between the BIA measurement intervals. The most common cause of admission was infection (n = 101 [37.1%]), followed by cardiovascular events (n = 60 [22.1%]), other medical causes (n = 47 [17.3%]), dialysis access-related problems (n = 16 [5.9%]), other surgical causes (n = 16 [5.9%]), malignancy (n = 15 [5.5%]), trauma (n = 11 [4.0%]), and degenerative diseases (n = 6 [2.2%]). BIA, bioelectrical impedance analysis; MHD, maintenance hemodialysis

**Table S1.** Body composition changes after hospitalization

|  | **General ward admission (n = 63)** | **P** | **ICU admission (n = 26)** | **P** |
| --- | --- | --- | --- | --- |
| BMI | −0.4 ± 1.2 | 0.015 | −0.2 ± 0.9 | 0.252 |
| FFMI | 0.1 ± 1.1 | 0.611 | 0.3 ± 1.4 | 0.266 |
| SMI | 0.0 ± 0.6 | 0.937 | 0.1 ± 0.7 | 0.428 |
| PBF | −1.6 ± 6.1 | 0.038 | −2.1 ± 6.4 | 0.115 |
| VFA | −4.2 ± 24.7 | 0.182 | −2.7 ± 29.8 | 0.645 |
| ECW/TBW | 0.2 ± 1.0 | 0.104 | 0.5 ± 0.01 | 0.033 |
| PhA | −0.1 ± 0.6 | 0.174 | −0.4 ± 0.9 | 0.029 |

Data are expressed as mean ± standard deviation.

BMI, body mass index; CI, confidence interval; ECW/TBW, ratio of extracellular water to total body water; FFMI, fat-free mass index; PBF, percent body fat; PhA, phase angle; SMI, skeletal muscle mass index; VFA, visceral fat area.
